# A Rare Case of Posterior Scleritis Masquerading as Acute Angle Closure Glaucoma: Highlighting the Diagnostic Value of Multimodal Imaging

**DOI:** 10.7759/cureus.85282

**Published:** 2025-06-03

**Authors:** Petros Asteris, Eleni Bagli, Chris Kalogeropoulos

**Affiliations:** 1 Department of Ophthalmology, University Hospital of Ioannina, Ioannina, GRC

**Keywords:** acute angle closure, glaucoma, posterior scleritis, scleritis, ubm

## Abstract

In this article, we present a rare case of posterior scleritis presenting as acute angle-closure glaucoma, highlighting the diagnostic challenges, clinical findings, and therapeutic approach, while emphasizing the importance of imaging modalities and detailed systemic evaluation. A 52-year-old, one-eyed, diabetic patient presented to the emergency department with mild ocular pain, photophobia, elevated intraocular pressure in the right eye, and a somewhat shallow anterior chamber. During examination, papilledema was noted. The patient was admitted for evaluation and management. During hospitalization, the patient’s condition deteriorated rapidly, with significant worsening in visual acuity and uncontrolled intraocular pressure elevation. Detailed ophthalmologic and systemic evaluations were performed, which included optical coherence tomography (OCT), B-mode ultrasonography (u/s B-mode), ultrasound biomicroscopy (UBM), fundus fluorescein angiography (FFA), and magnetic resonance imaging (MRI) of the eyes and orbit. Initial findings included optic disc edema, a shallow anterior chamber, and serous retinal detachment extending from the macula to the periphery. U/S B-mode revealed the characteristic “T-sign” indicative of posterior scleritis. MRI of the orbit and UBM revealed inward dislocation of the ciliary body and the presence of fluid in the suprachoroidal space of the right eye. Laboratory tests ruled out infectious and systemic autoimmune causes. Treatment for posterior scleritis led to significant improvement in visual acuity and intraocular pressure (IOP), along with gradual resolution of symptoms. Over nine months of follow-up, the patient remained stable. Posterior scleritis is a rare but important differential diagnosis in cases of acute angle-closure glaucoma with arduous IOP control. In such cases, imaging modalities such as u/s B-mode and UBM are crucial for diagnosis and management. Early recognition and an appropriate therapeutic approach can lead to favorable outcomes even in atypical presentations.

## Introduction

The term scleritis refers to inflammation of the sclera, the outer mantle covering the eye. Scleritis can be classified as anterior or posterior, depending on the part of the sclera that is involved, i.e., anteriorly or posteriorly of the rectus muscles' insertion, respectively.

Anterior scleritis is classified as 1. Diffuse anterior scleritis (widespread inflammation of the anterior sclera), 2. Nodular anterior scleritis (localized and tender scleral nodules), 3. Necrotizing anterior scleritis with inflammation, or 4. Necrotizing anterior scleritis without inflammation or scleromalacia perforans [1].

Posterior scleritis is classified as 1. Diffuse posterior scleritis (widespread inflammation of the posterior sclera) or 2. Nodular posterior scleritis [2]. Some cases of posterior necrotizing scleritis have also been reported [3,4]. Scleritis can also be unilateral or bilateral. Depending on the cause, scleritis can be classified as idiopathic, infectious, or non-infectious [5].

Although, quite often, the causative agent is not diagnosed at the time of first occurrence, it should be noted that scleritis has a strong correlation with systemic vascular diseases (granulomatosis with polyangiitis, polyarteritis nodosa) and rheumatoid arthritis [6]. It should also be noted that the symptoms that prompt patients to seek medical care are quite nonspecific (i.e., ocular pain, redness of the eye, tearing, and vision blurring). Of the two subtypes, posterior scleritis is by far the rarest and can present with a wide variety of clinical signs [7], presenting an arduous diagnostic challenge.

## Case presentation

We present herein an unusual case from our department with the use of the appropriate imaging materials concerning the patient. A 52-year-old man visited the emergency department of our clinic, complaining about mild pain in his right eye, accompanied by mild photophobia. These symptoms started about 10 days before the patient sought any medical assistance. The patient stated that although his symptoms had improved, they persisted, which is the reason he visited our department. The patient also stated that five days prior to his visit to our department, a private ophthalmologist had prescribed a combination of timolol and dorzolamide for his right eye due to a markedly elevated intraocular pressure (IOP) of 32 mmHg.

As far as the remainder of his medical history is concerned, the patient is a diabetic undergoing treatment with insulin. He also receives treatment for hypertension and dyslipidemia. He is also one-eyed, having lost his left eye 10 years ago due to acute postoperative endophthalmitis after pars plana vitrectomy for retinal detachment.

During his visit, a full routine examination was performed, with the following findings for his right eye: visual acuity (VA) was 0.8 decimal, IOP was 22 mmHg (measured with Goldmann applanation tonometry) with the use of timolol and dorzolamide, pupillary reaction was normal, the cornea was clear without the presence of edema, the anterior chamber was slightly shallow without any reaction, and the anterior chamber angle was narrow (Schaffer-Kanski Grade 2). Ocular motility was normal, with no pain. During undilated fundoscopy, optic disc edema was observed. Due to the patient's medical history, which made the diagnosis of sympathetic ophthalmia a possibility, a dilated fundus examination of both eyes was subsequently performed. No signs of choroiditis were observed. Taking into consideration the above-mentioned clinical findings, he was admitted to our clinic for further evaluation.

Based on our department's workup on ocular inflammations, a detailed laboratory investigation was carried out. This first diagnostic exploration included a full blood count and biochemistry assays, erythrocyte sedimentation rate (ESR), C-reactive protein (CRP), serum angiotensin-converting enzyme (SACE), as well as serological and immunological tests (Appendix). Although our patient was vaccinated in childhood with Bacille Calmette-Guérin (BCG), a purified protein derivative (PPD) skin test and a chest X-ray were performed to rule out tuberculosis. During admission, an optical coherence tomography (OCT) scan and u/s B-mode of the right eye were also performed, followed by fluorescein angiography. Magnetic resonance imaging of the brain and orbit was also scheduled.

**Figure 1:**
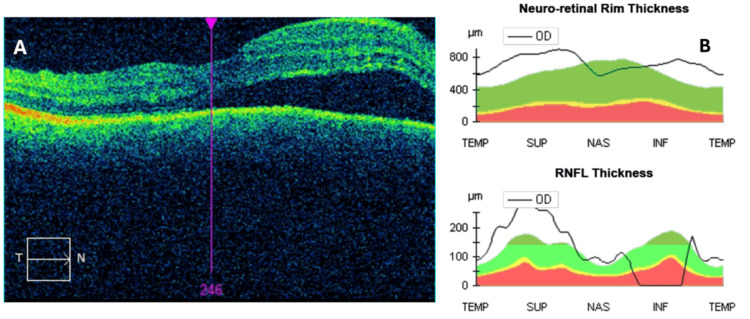
A: OCT of the macula showing serous retinal detachment, B: graphs of the right optic nerve showing elevated neuro-retinal rim and RNFL thickness (note the 0 μm measurement of the RNFL thickness at the inferior quadrant due to artifacts during acquisition). OCT: Optical coherence tomography, RNFL: Retinal nerve fiber layer

The patient was stable during the first two days of hospitalization. At this time, u/s B-mode didn’t show any significant findings; OCT of the optic nerve showed elevated retinal nerve fiber layer (RNFL) thickness due to the presence of edema along with serous retinal detachment at the macula (Figures 1A, 1B).

**Figure 2:**
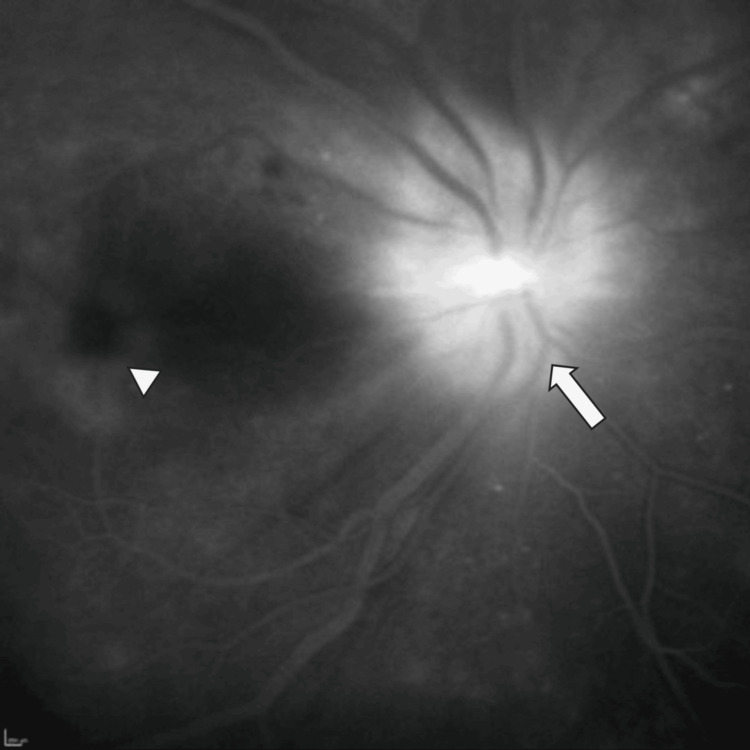
Late-phase fundus fluorescein angiography, showing optic disc edema (arrow) and the presence of fluid at the macula (arrowhead).

During this time, fundus fluorescein angiography (FFA) was performed, which corroborated the optic nerve edema and the presence of subretinal fluid at the macula (Figure 2). 

**Figure 3:**
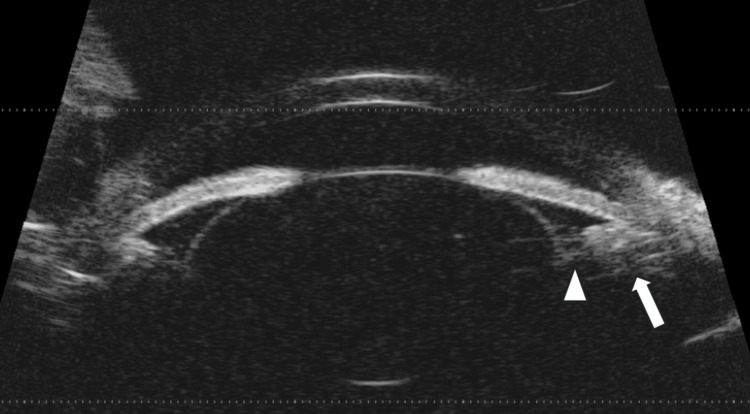
Ultrasound biomicroscopy of the right eye; note the forward dislocation of the iris-lens diaphragm and the shallow anterior chamber along with the inward dislocation of the ciliary body (arrow). The zonules are also visualized (arrowhead).

During the third day of hospitalization and for the next several days, the patient’s condition started to rapidly deteriorate. A significant reduction in VA was noted along with severe photophobia. Deep retrobulbar pain, mainly at night, also developed. The anterior chamber became markedly shallow, and control of IOP was arduous. An extensive examination of both eyes was also conducted during the escalation of symptoms. No signs of panuveitis were observed in either eye. During this time, ultrasound biomicroscopy (UBM) was performed, showing a very shallow anterior chamber, along with a slightly higher than normal anteroposterior crystalline lens diameter, forward dislocation of the iris-lens diaphragm, and inward dislocation of the ciliary body (Figure 3).

**Figure 4:**
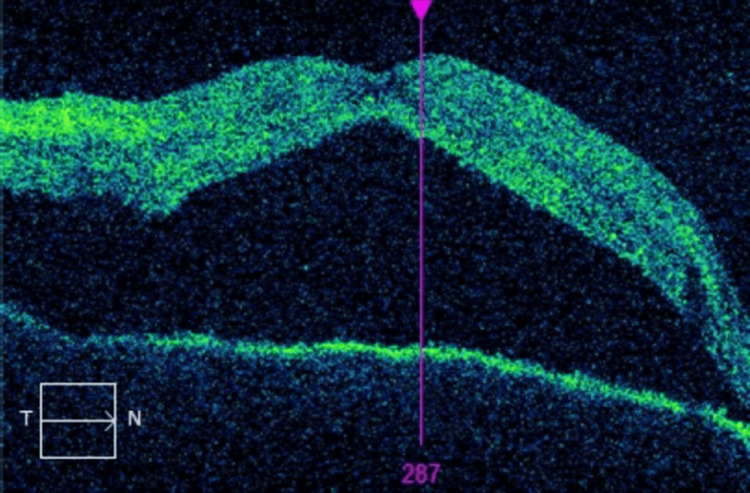
Second OCT of the macula with significant worsening of the serous retinal detachment. OCT: Optical coherence tomography

OCT of the macula, at this time, revealed a significant worsening of the subretinal fluid (Figure 4).

**Figure 5:**
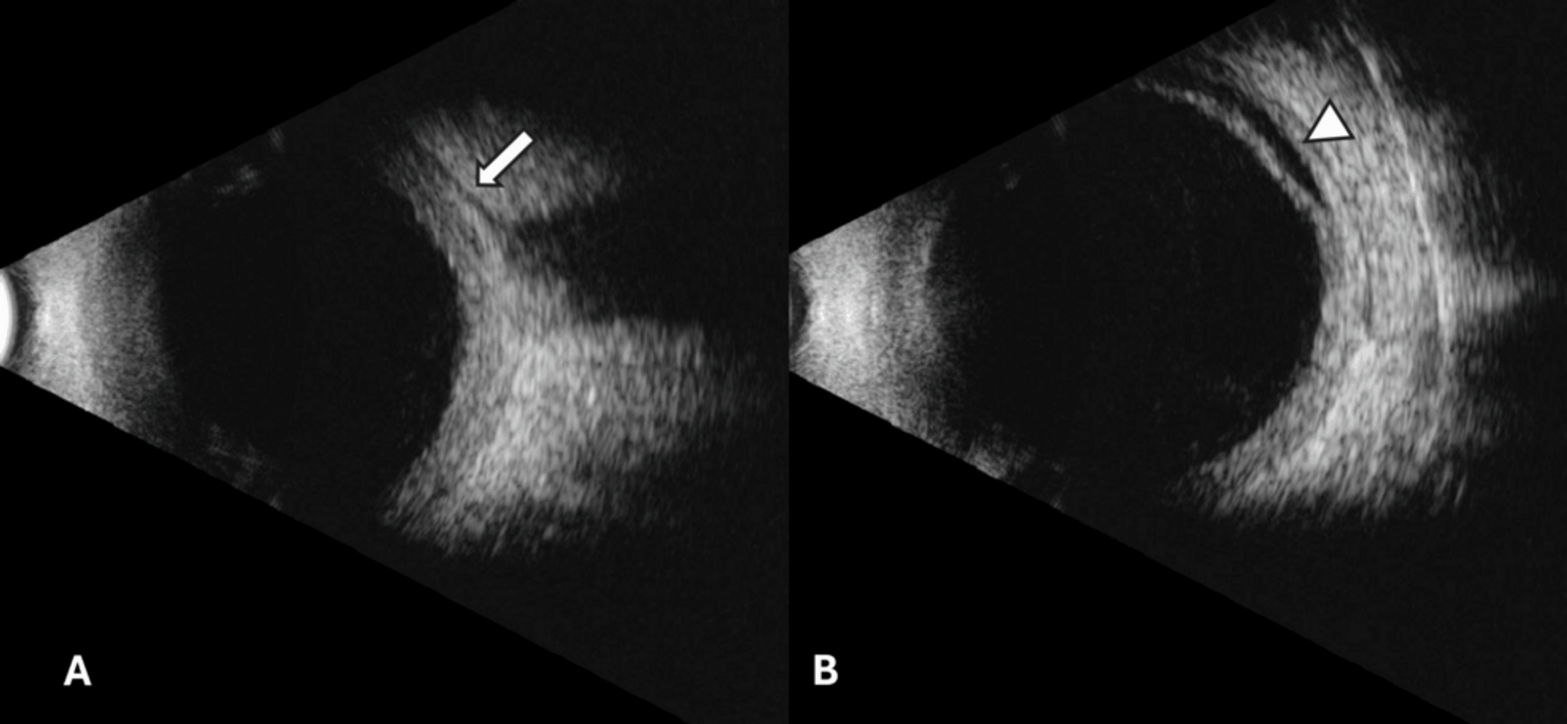
A: U/S B-mode at the optic nerve showing a partial "T-sign" (arrow). B: U/S B-mode of the peripheral retina, showing serous retinal detachment extending beyond the equator, towards the ciliary body (arrowhead).

A second u/s B-mode was performed, this time revealing a “T-sign,” indicative, in our case, of posterior scleritis. Serous retinal detachment, extending beyond the equator towards the ciliary body, was also noted (Figures 5A, 5B).

**Figure 6:**
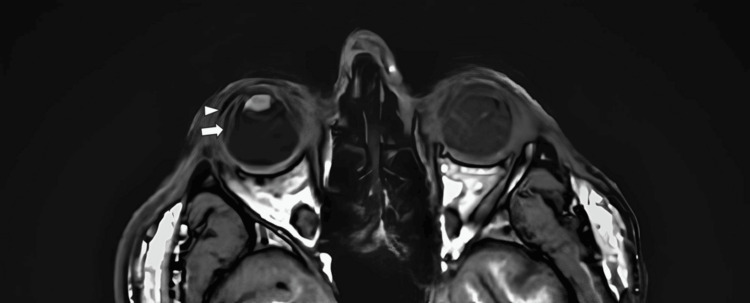
T1 MRI of the eye. Right eye: presence of fluid in the subretinal (arrow) and suprachoroidal (arrowhead) space. Left eye: Note the state of the left eye due to acute postoperative endophthalmitis.

At this time, the scheduled MRI of the orbit and brain was performed, revealing the presence of fluid in the suprachoroidal space of the right eye (Figure 6).

The results of the laboratory tests ordered during admission were normal. Therapy options were discussed with the patient. He did not wish to use steroids due to his difficulty in controlling diabetes. Taking into consideration the patient’s wishes, therapy was promptly started. The patient was administered topical dexamethasone drops five times daily, bromfenac drops twice daily, a combination of timolol/dorzolamide drops twice daily, atropine drops twice daily, oral acetazolamide 125 mg three times daily, 100 cc of intravenous mannitol twice daily, per os nonsteroidal anti-inflammatory drugs (NSAIDs), oral methotrexate 7.5 mg in the morning and 5 mg in the afternoon once per week, and oral folic acid (5mg) once per week. During the following seven weeks, when adequate control of IOP was achieved, oral acetazolamide and intravenous mannitol were discontinued, and the patient was discharged with instructions to follow the rest of the treatment and was scheduled for close follow-up. Currently, nine months after hospitalization, the patient does not receive any treatment and is stable without any symptoms. He has a VA OD of 0.9 decimal and an IOP of 12 mmHg and is closely monitored.

## Discussion

Before discussing specifics on posterior scleritis, a major differential diagnosis physicians should have in mind with patients with similar medical histories to our own is sympathetic ophthalmia. Sympathetic ophthalmia is an immune-mediated inflammatory response following ocular trauma or surgery. The eye that was injured or operated on is called the inciting eye, and the fellow, uninjured eye is called the sympathizing eye. It presents with asymmetric bilateral granulomatous panuveitis [8], signs that were not observed in our patient. Most cases (90%) present within a year of ocular injury, although the time of onset can vary greatly from weeks to decades [9].

Posterior scleritis is by far the rarest form of scleritis in adults. The incidence rate varies greatly among various retrospective studies, from 2% to 17.7% [10], and affects mainly women [11]. In contrast, posterior scleritis seems to be the most frequent form of scleritis in children [12-14].

The largest study we could find on posterior scleritis offers useful insights into a sample of 114 patients with posterior scleritis [15]. The majority of the cases presented with symptoms and signs similar to our case. However, of the 114 patients in the study, only eight presented with ocular hypertension, and none of them developed acute angle closure glaucoma due to ciliary body detachment and suprachoroidal effusion.

IOP elevation in posterior scleritis can be attributed to various factors. These include obstruction of the trabecular meshwork by inflammatory cells, adhesion of the cornea to the iris, or a delayed response to topical steroid drops. It should also be noted that an increased scleral thickness may oppress vortex veins and hinder the backflow of choroidal veins, which will facilitate choroidal congestion and ciliary body detachment [16].

In the study by Lavric et al., most cases (62.3%) presented as isolated, idiopathic posterior scleritis. The most frequently observed systemic associations included rheumatoid polyarthritis (12.28%), systemic lupus erythematosus (4.38%), and systemic vasculitis with positive pANCA, none of which could be identified in our patient.

The presence of the “T sign,” which was observed in our case, is generally considered pathognomonic for posterior scleritis. In truth, although posterior scleritis is by far the most common cause, other diseases have been rarely reported to exhibit the T-sign on ocular ultrasound (post-traumatic/post-surgical [17], orbital cellulitis [18], orbital tumors [19]/idiopathic orbital inflammatory syndrome [20]). None of these were identified in our patient.

The presence of the T-sign varies among various studies. Biswas et al. reported a “T sign” in about 25% of cases [21], while the aforementioned study by Lavric et al. reported a “T sign” in about 41.2% of their cases. In another study by Al Barqi M. et al., “T sign” was reported in 64.3% of posterior scleritis cases, although the sample consisted of only 14 cases [22].

Regarding therapy, in the study by Lavric et al., the most frequently used systemic therapeutic regimen for posterior non-infectious scleritis consisted of the administration of steroids. In severe cases, patients were administered 1 g of intravenous methylprednisolone for three days, with appropriate per os tapering. Other options include immunomodulatory drugs, antimetabolites (methotrexate-with folic acid-[23-25], mycophenolate mofetil), calcineurin inhibitors, alkylating agents, and biologics [26]. NSAIDs are used in conjunction with the other treatment options. Therapy should be tailored according to cause and patient profile. In our case, we opted for the use of methotrexate, taking into consideration the patient’s wishes and his medical history of diabetes. The median dose of methotrexate is 12.5 mg per week, ideally as an adjunct therapy to steroids or as steroid-sparing therapy [26], but can go as high as 25mg per week [27]. Blood tests were done monthly for toxicity monitoring. Atropine drops were used, as stated above, in order to push back the iris-lens diaphragm and the ciliary body to deepen the anterior chamber.

During our search for similar cases using PubMed and Google Scholar search engines, we found only a handful of cases of posterior scleritis presenting as acute angle closure glaucoma. Only one of them was bilateral [28]. One case was linked with antinuclear antibody (ANA) positivity, but a definite systemic diagnosis was not made [29]. One case was linked with Sturge-Weber syndrome [30]. One case was attributed to tuberculosis, for which the patient was already receiving treatment [31]. One case was attributed to psoriasis [32]. The rest of the cases were labeled as idiopathic, considering no clear systemic associations were found [33-35].

## Conclusions

In summary, posterior scleritis is a rare entity with a lot of systemic associations. It can manifest with a plethora of symptoms, which are nonspecific and require a detailed diagnostic exploration. Systemic disease, and especially vasculitis, should be considered. The use of the u/s B mode is invaluable in establishing a definite diagnosis. Cases like the one we presented are atypical but should be considered in the differential diagnosis of acute angle closure glaucoma, especially when managing IOP is difficult. The use of UBM, although not widely available, can be of great assistance in visualizing the anterior chamber and ciliary body and shedding light on the mechanism of IOP elevation, leading to proper therapeutic strategies.

## Appendices

**Table 1:** Tests ordered during patient hospitilazitaion

Systemic and Inflammatory Markers
Complete Blood Count (CBC) with differential
Erythrocyte Sedimentation Rate (ESR)
C-Reactive Protein (CRP)
Autoimmune and Rheumatologic Screening
Antinuclear Antibody (ANA)
Rheumatoid Factor (RF)
Anti-Cyclic Citrullinated Peptide (Anti-CCP)
Anti-Neutrophil Cytoplasmic Antibodies (ANCA)
Angiotensin-Converting Enzyme (ACE)
C3, C4
Serum IgA, IgE, IgG, IgM
Anti-dsDNA
Anti-cardiolipine IgM, IgG
Flow cytometry (peripheral blood sample)
Infectious Workup
Tuberculosis screening (Tuberculin Skin Test)
Syphilis serology
Hepatitis B and C serology
EBV IgG, IgM
CMV IgG, IgM
HSV1+2, IgG, IgM
VZV IgG, IgM
Borrelia burgdorferi IgG, IgM
Diabetes Monitoring
HbA1c (Glycated Hemoglobin)
Fasting Blood Glucose
Renal function tests
Others
Uric Acid
Complete biochemical assessment
